# Agility in Handball: Position- and Age-Specific Insights in Performance and Kinematics Using Proximity and Wearable Inertial Sensors

**DOI:** 10.3390/s25092728

**Published:** 2025-04-25

**Authors:** Pieter Heuvelmans, Alli Gokeler, Anne Benjaminse, Jochen Baumeister, Daniel Büchel

**Affiliations:** 1 Exercise Science and Neuroscience Unit, Department of Exercise & Health, Paderborn University, 33098 Paderborn, Germany; pieter.heuvelmans@upb.de (P.H.);; 2 Department of Human Movement Sciences, Faculty of Medical Sciences, University of Groningen, 9712 CP Groningen, The Netherlands; a.benjaminse@umcg.nl

**Keywords:** change of direction, neurocognition, cutting, prevention, injury

## Abstract

Handball is a dynamic team sport characterized by high agility requirements, which feature complex motor–cognitive demands. The ability to meet these demands is critical for performance in handball but remains under-represented in research. Existing studies highlight that cognitive demands can strongly interfere with motor behavior, particularly in dynamic sport-specific movement tasks. Furthermore, high motor–cognitive load is associated with risk of lower limb injury. Therefore, to gain insight in the mechanisms between movement and performance dynamics in the presence of cognitive demands, this study investigated the performance of elite handball players in a novel planned and reactive agility task. Four FitLight proximity sensors (FitLight Corp, Aurora, ON, Canada) recorded execution time. Nine Noraxon Myomotion wearable inertial sensors (Noraxon U.S.A. Inc., Scottsdale, AZ, USA) tracked the motion of the players’ trunk, pelvis, and lower extremities at 200 Hz. Execution time and kinematics were compared between adult and youth players and between different playing positions. Adult players demonstrated faster performance than youth players and exhibited differences in hip and knee flexion, potentially reflecting variations in acceleration and deceleration strategies. Backcourt players and wings demonstrated faster performance compared to pivots, who showed distinct patterns of hip, knee, and ankle flexion, possibly due to differences in body composition. These findings highlight the influence of motor and cognitive demands on agility performance and offer valuable insights into age- and position-specific differences among elite handball players. Furthermore, these findings support the use of wearable inertial sensors for the purpose of athlete evaluation. Future research should explore the implementation into athlete monitoring.

## 1. Introduction

Handball is a team sport with seven players per side, characterized by complex and intermittent activity profiles [1]. These profiles result from the dynamic play around and between the two goal areas, leading to frequent changes in activity. Players with substantial playing time (>50 min) typically perform about 16 jumps, 13 sprints, endure approximately 48 impacts, and engage in around 55 acceleration actions [2]. Agility, defined as a motor–cognitive skill involving the ability to quickly perceive information and alter speed or direction, is a crucial attribute in handball [3]. Handball-related research over the last decade set a major focus on the physical and neuromuscular determinants of performance [1]. Although agility is a critical aspect of handball, research has often neglected the cognitive and motor tasks involved [4]. Nevertheless, it is well known that the co-existence of cognitive tasks strongly interferes with motor behavior in dynamic sports-specific tasks requiring lower limb control such as jump landings or changes of direction [5,6]. Since high motor–cognitive load is known to be associated with high incidences for lower limb injuries [7,8], there is a need to develop appropriate tests for the assessment of motor–cognitive tests in handball players. Accordingly, these tests should integrate physical and cognitive demands that simulate the complex demands in handball [9].

Using technologies such as the FitLight system, execution times (i.e., the sum of reaction time and motor execution time) can be assessed to reflect the ability to perform motor–cognitive tasks. With portable setups, tasks can be tailored to sports-specific demands [10], allowing for ecologically valid testing of handball players [11]. In addition to being reliable [12], these tests also seem valid since they allow for the differentiation between defensive and offensive handball players when building ratios between planned and reactive movement tasks [9]. We suggest that incorporating cognitive cues into agility tasks may provide valuable insights into handball players’ motor–cognitive abilities, facilitating sports-specific performance evaluation and talent identification. Here, we suggest that faster agility times indicate improved motor–cognitive abilities, for instance, between youth and senior athletes [13].

Since agility evaluations typically express performance using execution times [14], little is known about kinematic changes in handball-specific agility tasks under reactive conditions. Previous research provided evidence of changes in knee kinematics during unplanned dynamic but isolated movements such as landings and sidestep cuts compared to planned control movements [15]. For instance, knee flexion at initial contact was found to decrease in sidestep cutting when performed under unanticipated conditions [16]. In turn, not only the change in execution time, but also the change in how athletes move may provide valuable insights into the ability of handball players to cope with the strenuous challenges in match play. Here, kinematic changes in motor–cognitively challenging conditions may allow for more tailored conditioning programs for the individual handball player.

Therefore, this study set out to investigate both performance and kinematic outcomes of a novel reactive task in elite handball players, providing insights into the ability of a player to cope with handball-related affordances. The primary aim was to describe changes in execution times in elite handball players between planned and reactive agility tasks. Based on the previous literature assuming that handball players’ physical performance differs depending on position [17] and age group [18], both factors were considered to test the discriminant validity of the developed test. This subtype of validity refers to the extent to which a test or measure accurately distinguishes between constructs or variables that are theoretically expected to be distinct [13]. The secondary aim was to explore the link between performance and movement by testing the kinematics for amplitude and timing differences for significantly different comparisons in execution time.

## 2. Methods

### 2.1. Participants

A total of 92 male handball players participated in this study: 66 adults and 26 youths (Table 1). A total of 53 adult players competed at the highest national level (Daikin Handball Bundesliga [GER], Håndboldligaen [DNK]), 13 at the second-highest level (First Division Denmark [DNK]), and all youth players competed at the highest U19 national level (Junioren-Bundesliga [GER]). In reference to the performance caliber classification of McKay et al. [19], 10 of the included athletes can be classified as Tier 1 (Worldclass) because of medals or individual trophies at Olympics or World Championships. The remaining 56 athletes can be classified into Tier 2 to Tier 3 because they were competing in international- and national-level competitions with their clubs and national teams. Informed consent was obtained from all participants prior to their involvement in the study. Participants were provided with detailed information about the study’s purpose, procedures, potential risks, and the voluntary nature of participation. They were informed that their participation was voluntary and that they could withdraw from the study at any time without consequence. Consent forms were signed by all participants to confirm their understanding of the study details and agreement to participate. The study adhered to ethical guidelines and was approved by Institutional Review Board (IRB).

### 2.2. Experimental Design

**Figure 1:**
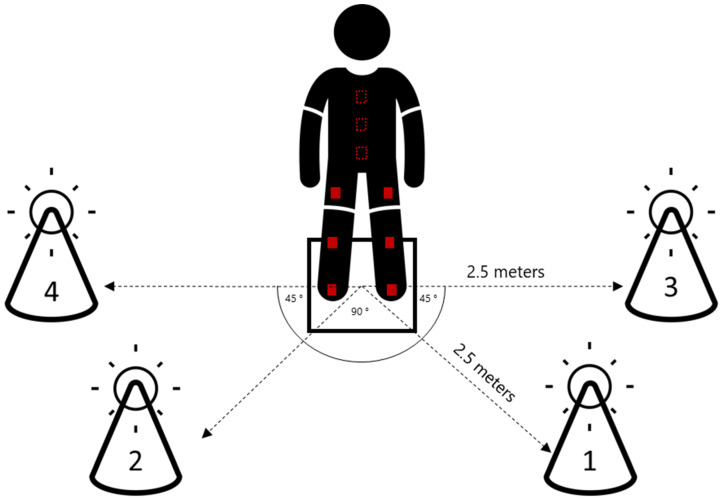
Graphical representation of task setup. All lights are positioned on cones 2.5 m away from the starting square. Participants were asked to run as fast as possible to the initiated light and turn it off by swiping the hand within 40 cm above the light. To quantify kinematics, all subjects wore IMUs placed at the feet, shanks, thighs, pelvis, T12, and C7 (red boxes/squares).

The study was designed as a cross-sectional performance assessment to evaluate elite handball players’ reactive ability and lower extremity kinematics. The FitLight system (FitLight Corp, Aurora, ON, Canada), a wireless reaction training system that measures execution time, was used for the assessment. The system comprises a set of LED lights arranged on top of 38 cm-tall cones in a trapezoid layout, with four lights programmed to activate in specific sequences. The height of the cones was chosen to demand substantial knee and hip flexion, a key aspect of movement quality during cutting maneuvers [20]. Two lights were placed at 45-degree angles relative to the starting position, while the other two lights were positioned perpendicular (90-degree angles) to the starting position, located directly to the left and right of the participant. All lights were situated 2.5 m away from the starting position, requiring participants to deactivate them promptly using their hands in response to the activation sequences. The proximity setting was set to 40 cm. Nine Noraxon inertial measurement units (IMUs) were used to record kinematics at 200 Hz. The IMUs were attached to the body at the feet, shanks, thighs, pelvis, T12, and C7, using proprietary straps and according to the manufacturer’s instructions (Noraxon MyoMotion Hardware User Manual, Noraxon U.S.A. Inc., Scottsdale, AZ, USA). Previous research has reported on the exact IMU placement in addition to validating the use of this setup for the collection of sagittal joint angles at the hip, knee, and ankle during change-of-direction movements [21]. An overview of the experimental setup can be found in Figure 1.

### 2.3. Procedure

The participants were given time for a self-selected warm-up in preparation for the agility tests, with a minimum of 10 min. Participants completed ten rounds, divided into two conditions, starting with planned movement tasks and followed by unplanned reactive movements. The planned movement task was as follows: A total of 5 rounds were conducted with a predetermined, consistent sequence of 10 lights (i.e., 1-2-1-2-1-2-1-2-3-4). The lights were numbered 1 to 4 for front left, front right, lateral left, and lateral right, respectively (Figure 1). The sequence remained constant across these five rounds, allowing players to anticipate the next light based on prior knowledge. The reactive movement task was as follows: A total of 5 rounds were conducted with a random sequence of 10 lights, where the activation order of the lights was unpredictable for the participants. This condition was designed to assess the players’ ability to respond to unplanned stimuli. The interval between subsequent light activations was set to 2 s.

### 2.4. Data Collection

For each repetition, the FitLight system recorded the time taken by the player to deactivate each light. As a dependent variable, median execution times for both the planned and reactive conditions were extracted. During each repetition, from light activation to light deactivation, lower extremity joint kinematics were recorded and processed with proprietary software (Noraxon MR3, version 3.20.02). Kinematic data were selected for the leading leg (i.e., the ipsilateral leg to the light) for light numbers 1 and 2 as these were designed to elicit a diagonal (i.e., 45-degree) acceleration and deceleration movement, which is expected to provide insights into knee stability relevant for performance as well as return-to-play scenarios. These types of movements have previously been investigated during agility assessments [12,22]. Light numbers 3 and 4 were included as a distractor to minimize anticipatory behavioral strategies during the reactive task. Due to the limited number of repetitions collected for light numbers 3 and 4, and because the movement directions are inherently different, they were excluded from analysis. Kinematic data were time-normalized to 101 equally spaced data points with linear registration using interpolation. To allow for the simultaneous assessment of amplitude and timing differences in the kinematic waveform signals, nonlinear registration using warp functions was conducted, producing coupled amplitude vectors and displacement fields [23]. All kinematic analyses were conducted in custom Python scripts using the *nlreg1d* and *spm1d* packages (Python 3.8.19).

### 2.5. Statistical Analysis

Data were tested for normality using the Shapiro–Wilk test. Comparisons of age, height, and weight between age groups and playing positions were conducted using Mann–Whitney U and Kruskal–Wallis H tests, respectively. Kruskal–Wallis H tests were followed by Dunn’s tests for post hoc pairwise comparisons. Differences in execution time data were tested using a two-way repeated-measures ANOVA with age group (adult, youth) and playing position (backcourt, pivot, wing) as between-subjects factors and condition (planned, reactive) as a within-subjects factor. The assumption of homogeneity of variances was tested with Box’s M Test of Equality of Covariance Matrices. The alpha level was set to 0.05 for all tests. Post hoc pairwise comparisons were performed with Bonferroni correction. Partial eta squared was interpreted as negligible, small, medium, or large for values <0.01, <0.06, <0.14, or ≥0.14 [24]. Main effects of age group and playing position were intended to display the discriminatory validity of the handball-specific agility test. Based on any identified differences in execution time, statistical nonparametric mapping (SnPM) would be conducted on the kinematic data [23]. Kinematic data were tested for amplitude and timing differences between conditions, age group, and playing positions. For each comparison, a multivariate Hotelling’s test was conducted on the coupled amplitude vectors and displacements fields. T-tests were conducted as a post hoc procedure with Bonferroni correction on the amplitude vectors and displacements fields, respectively.

## 3. Results

**Figure 2:**
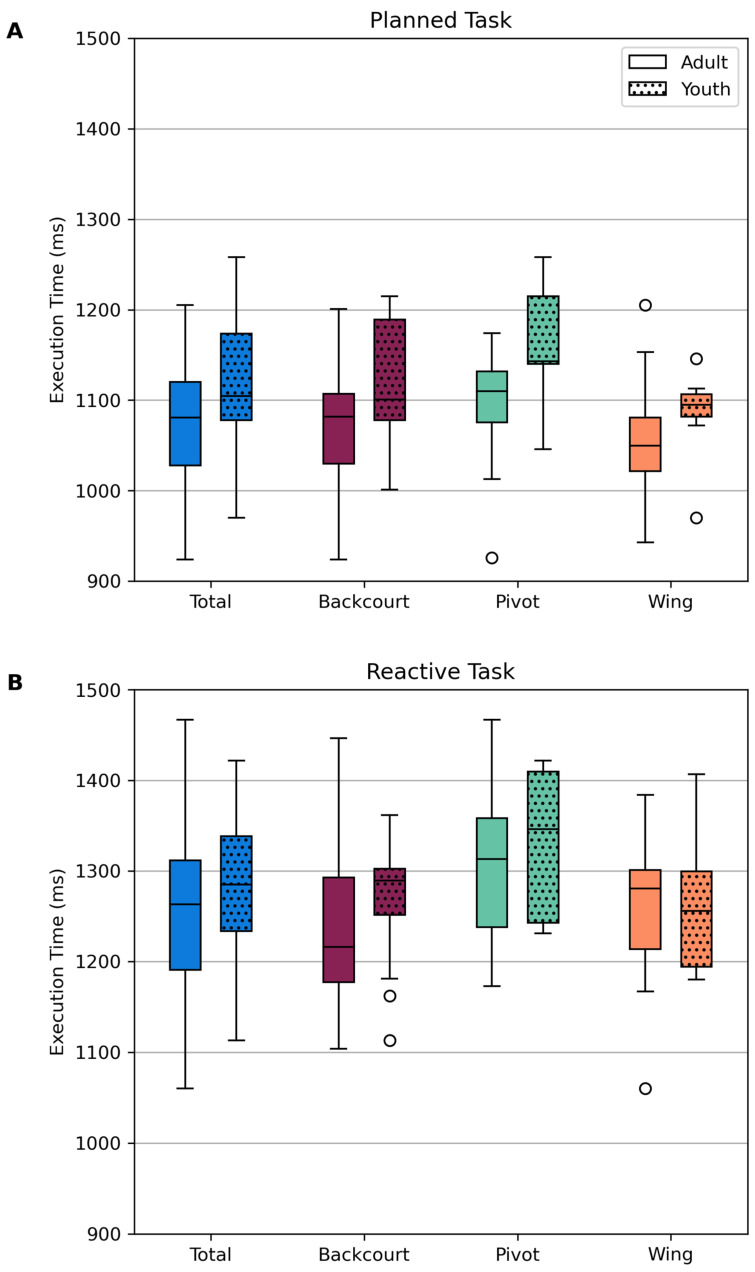
Execution time distributions for the (**A**) planned and (**B**) reactive tasks, per age group and playing position.

At the time of testing, the adult handball players were significantly older (median difference = 7 years, *p* < 0.001), taller (median difference = 6.5 cm, *p* = 0.025), and heavier (median difference = 12.15 kg, *p* < 0.001) than the youth players (Table 1). Pivots were significantly taller when compared to backcourt players (median difference = 4.5 cm, *p_adj_* < 0.001) and wing players (median difference = 9.5 cm, *p_adj_* < 0.001) (Table 2). Additionally, pivots were also significantly heavier than backcourt players (median difference = 14.9 kg, *p_adj_* < 0.001) and wing players (median difference = 22.9 kg, *p_adj_* < 0.001). Backcourt players were significantly taller (median difference = 5 cm, *p_adj_* = 0.032) and heavier (median difference = 8 kg, *p_adj_* = 0.030) than wings. A two-way repeated-measures ANOVA on execution time data identified main effects for condition (planned, reactive), age group (adult, youth), and playing position (wing, backcourt, pivot) (Figure 2). Execution times were shorter during the planned task than in the reactive task, with a mean difference of 182 milliseconds (*F*(1,86) = 344.41, *p* < 0.001, η^2^_p_ = 0.80). Adults were faster than youths, with a mean difference of 33 milliseconds (*F*(1,86) = 4.52, *p* = 0.036, η^2^_p_ = 0.05). Pivots were slower than backcourt and wing players (*F*(2,86) = 4.07, *p* = 0.020, η^2^_p_ = 0.09), with mean differences of 45 milliseconds (*p* = 0.029) and 50 milliseconds (*p* = 0.033), respectively. No significant interaction effects were found (*p* > 0.05).

Based on identified differences in execution time with regard to condition, age group, and playing position, SnPM analyses were performed.

### 3.1. Condition

**Figure 3:**
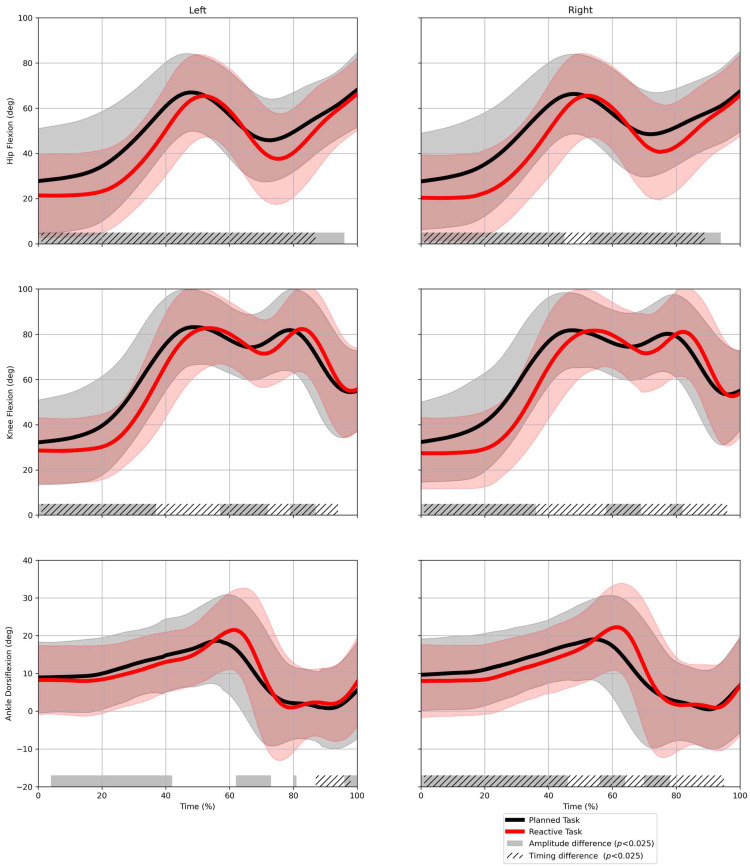
Kinematic comparison between planned and reactive tasks using statistical nonparametric mapping. Data are pooled over age groups and playing positions. Waveforms represent average hip/knee/ankle flexion for the ipsilateral leg in the movement to the left (light #1) and to the right (light #2).

In the reactive condition, significant timing differences compared to the planned condition were found across all joints (Figure 3). These differences indicate that movement responses were delayed during the reactive task. SnPM also identified significant amplitude differences for all joints between the conditions (Figure 3). During the reactive task, players exhibited less hip flexion in two parts of the movement task, between approximately 0–40% and 60–90% of normalized time. Knee flexion was also significantly lower between approximately 0–35% and 60–70% of the movement response. Ankle dorsiflexion showed amplitude differences similar to the hip and knee, but also increased peak at around 65% of the movement response.

### 3.2. Age Group

**Figure 4:**
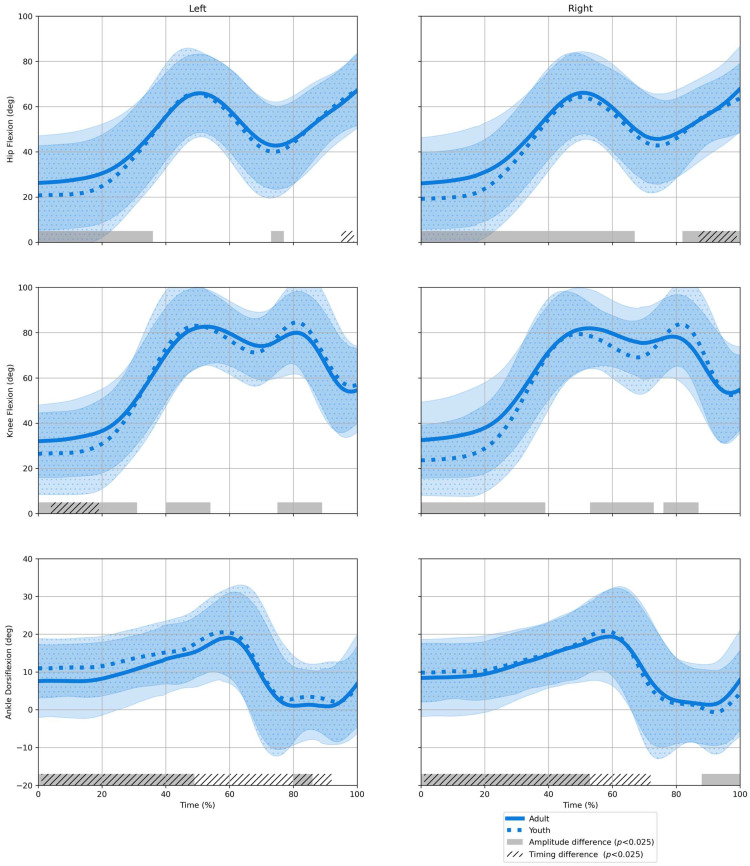
Kinematic comparison between adult (*N* = 66) and youth players (*N* = 26) using statistical nonparametric mapping. Data are pooled over conditions and playing positions. Waveforms represent average hip/knee/ankle flexion for the ipsilateral leg in the movement to the left (light #1) and to the right (light #2).

When comparing age groups, very few significant timing differences were identified at the hips and knees (Figure 4). Only the sagittal movement at the ankle appears to feature prevalent timing differences. Between the age groups, several significant amplitude differences were found across all joints (Figure 4). Youth players moved with less hip and knee flexion between 0 and 30%, whereas they showed greater peak knee flexion at around 80% of the movement response.

### 3.3. Playing Position

**Figure 5:**
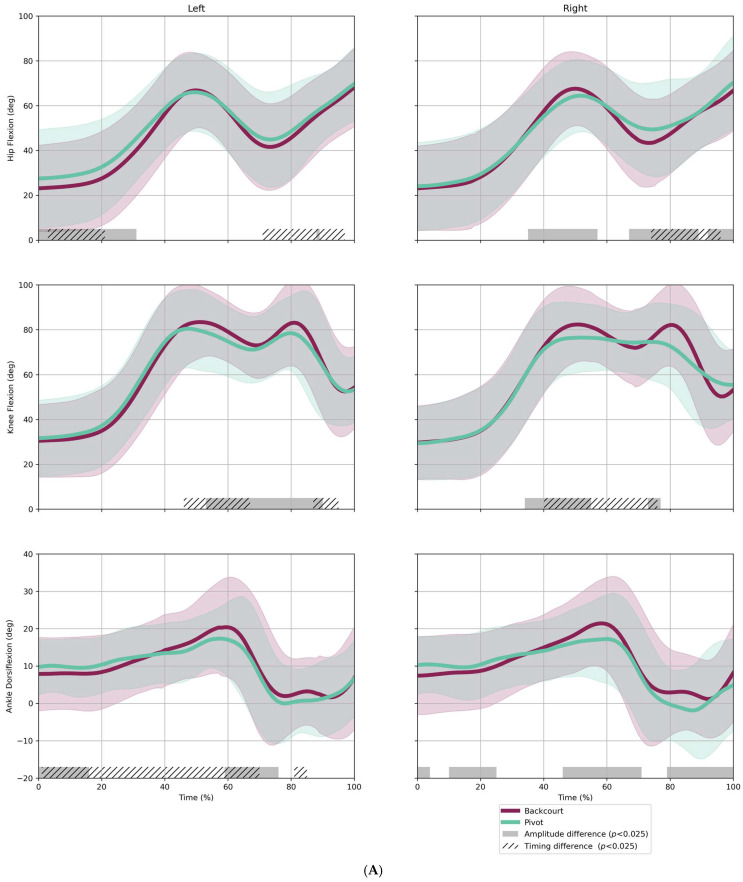

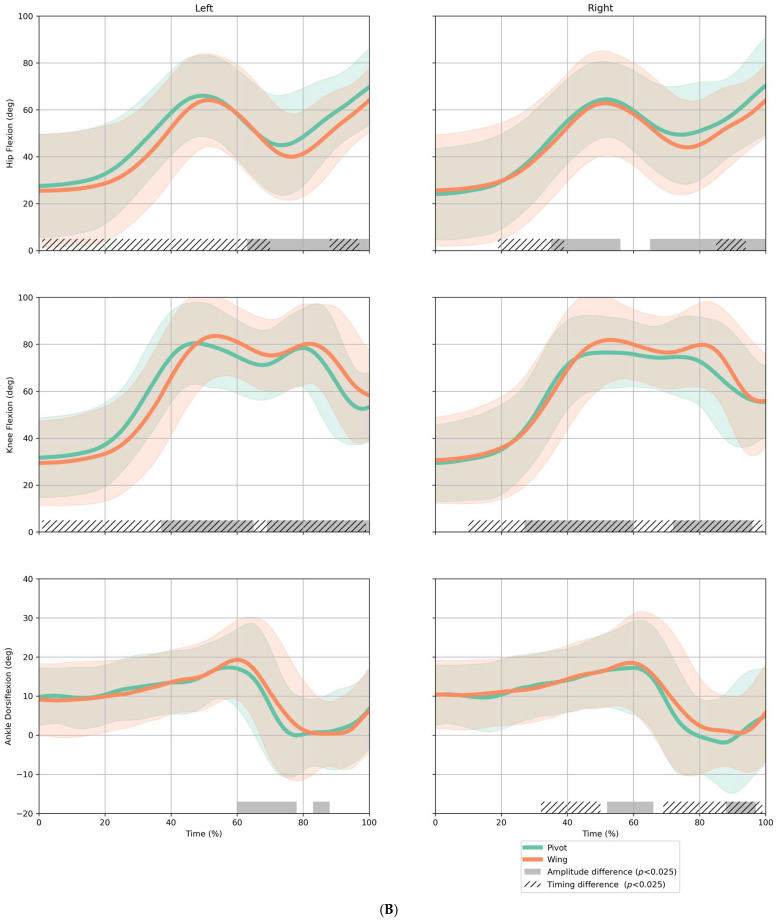
Kinematic comparison between (**A**) backcourt (*N* = 47) and pivot (*N* = 20); and (**B**) wing (*N* = 25) and pivot (*N* = 20), using statistical nonparametric mapping. Data are pooled over conditions and age groups. Waveforms represent average hip/knee/ankle flexion for the ipsilateral leg in the movement to the left (light #1) and to the right (light #2).

When comparing backcourt players to pivots (Figure 5A), significant timing differences were found for the hips around 70–90% and for knees between approximately 45 and 65% of the movement task. Moreover, SnPM found significant amplitude differences across all joints. Pivots moved with more hip flexion and ankle dorsiflexion, but with less knee flexion than backcourt players. When comparing wing players to pivots, significant timing differences were very prevalent in the hips and knees (Figure 5B). SnPM also identified significant amplitude differences, with pivots moving with more hip flexion than wing players around 65–100% of the movement response. In turn, wing players moved with more knee flexion in two parts of the movement, around 40–60% and 70–100% of normalized time. Wing players also showed greater ankle dorsiflexion than pivots, especially for the peak at around 60% of the movement response.

## 4. Discussion

This study assessed the execution time and kinematic performance of elite handball players in planned and reactive agility tasks. The main objective was to investigate how execution times and kinematics change for the reactive task between the different age groups and playing positions, and three main findings emerged. First, during the reactive condition, the handball players executed the task significantly slower and moved with significant amplitude differences in the lower extremity joints, including less hip flexion. Second, the adult handball players were significantly faster than the youth players, and the adults moved with significant amplitude differences compared to the youth players, notably in hip and knee flexion. Third, pivots were slower in performing the task than backcourt and wing players, and pivots also showed significant differences in the movements of their lower extremity joints compared to the other playing positions, including less knee flexion.

### 4.1. Condition

Analysis of execution time identified a main effect of condition, with significantly higher execution times during the reactive task compared to the planned task. This difference embodies the impact of random stimuli presentation on task execution. The planned task featured a predefined sequence of stimuli, providing a relatively high level of control to the player [25]. Switching the light sequence from planned to random increased motor–cognitive load, which slowed down task performance. This result is consistent with previous research on agility or changes of direction [9,22,26]. From a neurocognitive perspective, the observed increase in execution times during the reactive task reflects the added cognitive demands imposed by the unpredictable nature of the stimulus presentation and highlights the interplay between visual processing, cognitive decision-making, and motor responses in handball-specific situations.

SnPM analysis of the kinematic data found timing differences between the conditions, indicating that players moved with significantly delayed movements in the reactive task. These timing differences are most pronounced in the hip and knee joints, from approximately 0% to 90% of the movement response (Figure 3). The increased execution time in the reactive task, in addition to the prevalent timing effects, suggests that there was a change in *reaction time* (i.e., the time from stimulus presentation to movement initiation). This finding is consistent with that of previous research that identified a delayed reaction time to initiate movement when stop-signals were introduced to a simple foot-reaction tasks [27], but also a more complex lateral stepping task [28].

### 4.2. Age Group

This study also identified a main effect of age group. The adult handballers were faster than the youth players. This indicates that, despite being physically larger in average height and weight (Table 1), the adult players could outperform the youth players in the agility tasks. This finding may potentially be related to greater muscle mass in the adult group, as age has been associated with increased muscle mass in elite handball players [29]. Considering quick and reactive changes of direction as a key demand in handball [2], the discriminatory validity of the developed agility test can be confirmed, since players with higher playing level outperformed those from lower level. This finding is in line with previous research in high-level handball that described how adult players run faster, jump higher, throw faster, and have better aerobic fitness [18]. When implemented as a screening or monitoring tool alongside other diagnostic tests, this protocol may therefore prove effective for talent selection or performance optimization in elite handball.

SnPM analysis of the kinematic data found differences in the movements between the age groups (Figure 4). The adult handball players, who were on average faster in task execution than their youth counterparts, moved with no prominent timing difference in either the hip or the knee joints. This finding indicates that adults did not have substantial performance gains by initiating their movements earlier; otherwise, the SnPM results would have resembled the persistent timing differences between conditions (Figure 3). Given the minimal timing differences and the linear registration procedure (i.e., time normalization), the superior performance of adult players is more likely due to rapid movement execution rather than faster movement initiation. These results further support the association between handball player age and speed [18]. SnPM analysis of the kinematic data between the age groups also identified significant amplitude differences in the joint movements. Overall, the adult players moved with increased hip and knee flexion early in the movement suggesting that adults adopted a starting posture that was more crouched. Such adaptations of body posture have been reported to be beneficial for higher ground reaction forces, which may allow for a faster movement in the task due to enhanced acceleration [30]. However, adults showed lower peak knee flexion than the youth players later in the movement, indicating that the youth players employed more of the knee flexion range of motion of their leading leg during deceleration. Existing research recognizes the role of a *penultimate* step in decelerating for a change-of-direction movement and the effect of reduced planning time on reactive conditions [31]. Together, these findings potentially hint at differences in acceleration/deceleration strategies between youth and adult players. Further work is needed to better understand age-related differences in movement strategies in high-level handball.

### 4.3. Playing Position

Pivots were slower in executing the task than backcourt and wing players, regardless of condition. While previous studies compared agility performances between offence and defence players, no study so far has compared agility performance in a large sample of handball players according to playing positions. The execution time-related differences indicate that backcourts and wings outperform pivots in quick changes in direction, in both planned and reactive scenarios.

In handball, the role of a pivot is to travel along the opponent’s goal area line to create openings for attack on goal. Here, pivots typically need to defend their position in front of the goals and move in smaller spaces compared to wings and backcourts [32]. This role features a lot of physical contact with the defenders; hence, elite pivots spend a significant amount of time in high-intensity activities with substantial strength requirements [33,34]. Consequently, pivots usually have higher body mass than other playing positions (Table 2) [35], and they are also reported to have greater upper body strength when assessed by one-rep-max in bench press [17]. In contrast, backcourt or wing players’ tasks involve more running and sudden deceleration [2]. Previous research revealed that wings generally cover the longest distance at high speed or by sprint [33,36] while backcourt players have the highest overall running pace and center backs in particular endure more high-intensity decelerations [33,37]. Since our test particularly tested the ability to move quickly, the superior performance of backcourts and wings may, in part, be based on the position-specific running demands. Furthermore, backcourt players typically face higher cognitive demands in game situations, as they perform more passes, more shots, and more jumps per game [38]. The greater exposure to situations requiring motor–cognitive decision-making may contribute to their ability to perform the investigated agility tasks more quickly. These inherent differences between playing positions from a motor–cognitive perspective might explain why pivots were slower to perform the agility task than backcourt and wing players. Following the theory of affordances [39], players from different playing positions may consequently have distinct opportunities for actions. These chronic affordances may over time lead to position-specific adaptations of motor–cognitive capabilities. Researchers have previously argued that the differences in on-court demands are reflected in physiological and physical differences between playing positions, and, therefore, they surmise that strength and conditioning practices should be individualized and position-dependent [17]. Similar observations were made in basketball players, where the individuals also face highly position-specific demands [40].

In addition, SnPM analysis revealed that playing positions also showed significant differences in their movement behavior. The most striking finding reveals that pivots execute movements with less knee and ankle flexion but increased hip flexion compared to backcourt and wing players. The first suggests that pivots move with a distinct movement strategy that is less crouched or low to the ground. Since a less crouched position opposes the generation of ground reaction forces, this posture may contribute to reduced agility performance in pivots [30]. It might be speculated that body composition aspects such as the height, weight, and mobility of the pivots may require an adaptation of movement behavior. Further, our tests suggest that pivots employ more hip hinge when reaching for the light in the final part of the movement. Considering the standardized height of the agility task targets, the increased hip hinge may result from the differences in body height of 4.5 to 9.5 cm between pivots, wings, and backcourts. These findings highlight the influence of handball positions and physical characteristics on agility performance. Coaches and trainers should consider these positional differences when designing training programs, tailoring exercises to improve agility and movement efficiency according to each player’s role and body type.

### 4.4. Limitations

The light-based stimuli used in this investigation were not sport-specific; when playing a match, handball players typically respond to more complex stimuli like changes in body posture of the opponent. Previous research has shown that agility assessments using light-based and sport-specific stimuli test different qualities of team sport athletes [41]. The light-based stimuli obviously lack any information used for affordance perception; however, when compared to more traditional arrow stimuli on a TV, the lights do present a greater perceptual challenge to the player. Due to their different positions—diagonally in front and in the periphery on both sides—the lights prompt different visual behavioral strategies in the players. The choice of stimuli for the current study was based on standardization and feasibility concerns. Future research should attempt to improve the athlete–environment coupling by designing experiments with sport-specific stimuli. Here, one could also consider the implementation of targets at different heights to better simulate the diversity of handball-specific movements such as passing from shoulder height or receiving passes below hip height.

The execution time measures in this study did not capture the time it took to return to the starting position. The objective to quickly return to a starting position is relevant for performance in handball, especially during defence when players work to maintain a barrier at the six-meter line. Future investigations should therefore consider designing protocols that measure both the outward movement and the return phase.

The current study presented a novel agility protocol for the assessment of planned and reactive movements in handball players. However, it is important to acknowledge that there is no universally valid or standardized method for assessing agility that applies to all athletes or situations [42]. Agility is a complex, multifaceted skill that varies depending on the sport, the demands of the specific position, the intention of the player, and the context in which it is being performed (e.g., evading an opponent, intercepting a pass, or setting up an attempt at goal). Therefore, researchers should understand the specific context and objectives of the agility assessment before selecting the most appropriate testing protocol.

In comparing kinematics between age groups, significant timing differences were identified for ankle dorsiflexion (Figure 4). However, the kinematic waveforms give no clear indication of this timing difference nor its direction. This paradoxical finding may be due to the methodology used. The SnPM analysis in this study used nonlinear registration with warp functions. Warp functions rely on waveform geometry to produce displacement fields that quantify potential timing differences [43]. In this instance, the ankle dorsiflexion features few pronounced slope characteristics (i.e., peaks and valleys) and therefore may have affected the accuracy of the warping procedure.

## 5. Conclusions

This study highlights key kinematic and performance differences among elite handball players when executing agility tasks under planned and reactive conditions. Adult players were faster than youth players and moved with different hip and knee flexion, potentially hinting at differences in acceleration/deceleration strategies. Backcourt players and wings were faster than pivots, who moved with distinct hip, knee, and ankle flexion which are possibly related to differences in body composition. The findings emphasize the impact of motor and cognitive demands on agility performance and provide valuable insights into age and playing position-dependent differences in elite handball players. Moreover, these findings reinforce the value of wearable inertial sensors in assessing athlete performance. Future research should further explore the integration of movement quality data into comprehensive athlete monitoring, assessing their effectiveness in tracking of performance and screening for injury risk. Here, single-sensor applications may also provide valuable information into acceleration and deceleration capacities of the individual in handball-specific scenarios [44].

## Figures and Tables

**Table 1:** Demographics of the study population, per age group.

	Adult	Youth		
	*N* = 66	*N* = 26	*U*	*p*
Age (yrs)	24.00 (22.00–27.00)	17.00 (17.00–18.00)	1716.00	<0.001 *
Height (cm)	192.50 (189.00–196.00)	186.00 (183.00–196.25)	1116.00	0.025 *
Weight (kg)	95.90 (89.25–104.67)	83.75 (77.20–96.17)	1245.50	<0.001 *

Note: Values are median (Q1–Q3). *U*: Mann–Whitney U test result, *p*: *p*-value. All youth participants practiced in the U19 handball division. Asterisks indicate significant differences (*p* < 0.05) between age groups.

**Table 2:** Demographics of the study population, per playing position.

	Backcourt	Pivot	Wing		
	*N* = 47	*N* = 20	*N* = 25	*H*	*p*
Age (yrs)	22.00 (18.00–25.00)	24.00 (18.75–27.25)	23.00 (18.00–24.00)	1.49	0.475
Height (cm)	192.00 * (185.00–196.00)	196.50 * (195.00–199.25)	187.00 * (182.00–191.00)	28.34	<0.001
Weight (kg)	94.00 * (84.25–100.25)	108.90 * (99.35–116.08)	86.00 * (80.00–90.90)	36.29	<0.001

Note: Values are median (Q1–Q3). *H*: Kruskal–Wallis test result, *p*: *p*-value. Asterisks indicate significant differences (*p_adj_* < 0.05) following Dunn’s test for post hoc pairwise comparison with Bonferroni correction.

## Data Availability

The data presented in this study are available on reasonable request. The data are not publicly available due to privacy restrictions.
